# The constructive function of apoptosis: More than a dead-end job

**DOI:** 10.3389/fcell.2022.1033645

**Published:** 2022-12-13

**Authors:** Gabriel Krasovec, Helen R. Horkan, Éric Quéinnec, Jean-Philippe Chambon

**Affiliations:** ^1^ Centre for Chromosome Biology, School of Natural Sciences, University of Galway, Galway, Ireland; ^2^ ISYEB, Institut de Systématique, Evolution et Biodiversité, Sorbonne Université, CNRS, MNHN, Paris, France; ^3^ CRBM, University of Montpellier, CNRS, Montpellier, France

**Keywords:** apoptosis, caspase, cell fate, cell behavior, signaling center

## Introduction

Apoptosis is a form of regulated cell death defined by a characteristic and conserved set of morphological features (Kerr et al., 1972) that depend on a protease family named caspases (Hengartner, 2000). From these characteristics, apoptosis has been identified in most metazoan phyla, including vertebrates, insects, mollusks, and cnidarians (Lockshin and Williams, 1964; Yuan et al., 1993; Accordi and Chimenti, 2001; Chambon et al., 2002; Sokolova et al., 2004; Vega Thurber and Epel, 2007; Pirger et al., 2009; Krasovec et al., 2021b). Apoptosis has been considered to have merely a direct function in the elimination of structures or unwanted cells, which could be damaged, obsolete, or redundant (the so-called destructive function of apoptosis—DFA). The DFA is well studied and mechanistically well understood and characterized in various morphogenetic processes, such as embryogenesis, metamorphosis, and homeostasis, in addition to being the historic and dominant vision of the function of cell death (Jacobson et al., 1997). For example, the DFA allows the elimination of obsolete tissue such as the interdigital tissues during the embryogenesis of some tetrapod vertebrates (Hernández-Martínez et al., 2009). The deletion of larval tissues during metamorphosis is also a classic illustration of the DFA, such as in ascidians or frogs (Accordi and Chimenti, 2001; Chambon et al., 2002; Jeffery, 2002). The DFA is equally important for tissue homeostasis in adult animals, maintaining the correct number of cells in all organs in a dynamic fashion; for example, the gonad size in rodents changes according to reproduction periods (Furuta et al., 1994).

**FIGURE 1:**
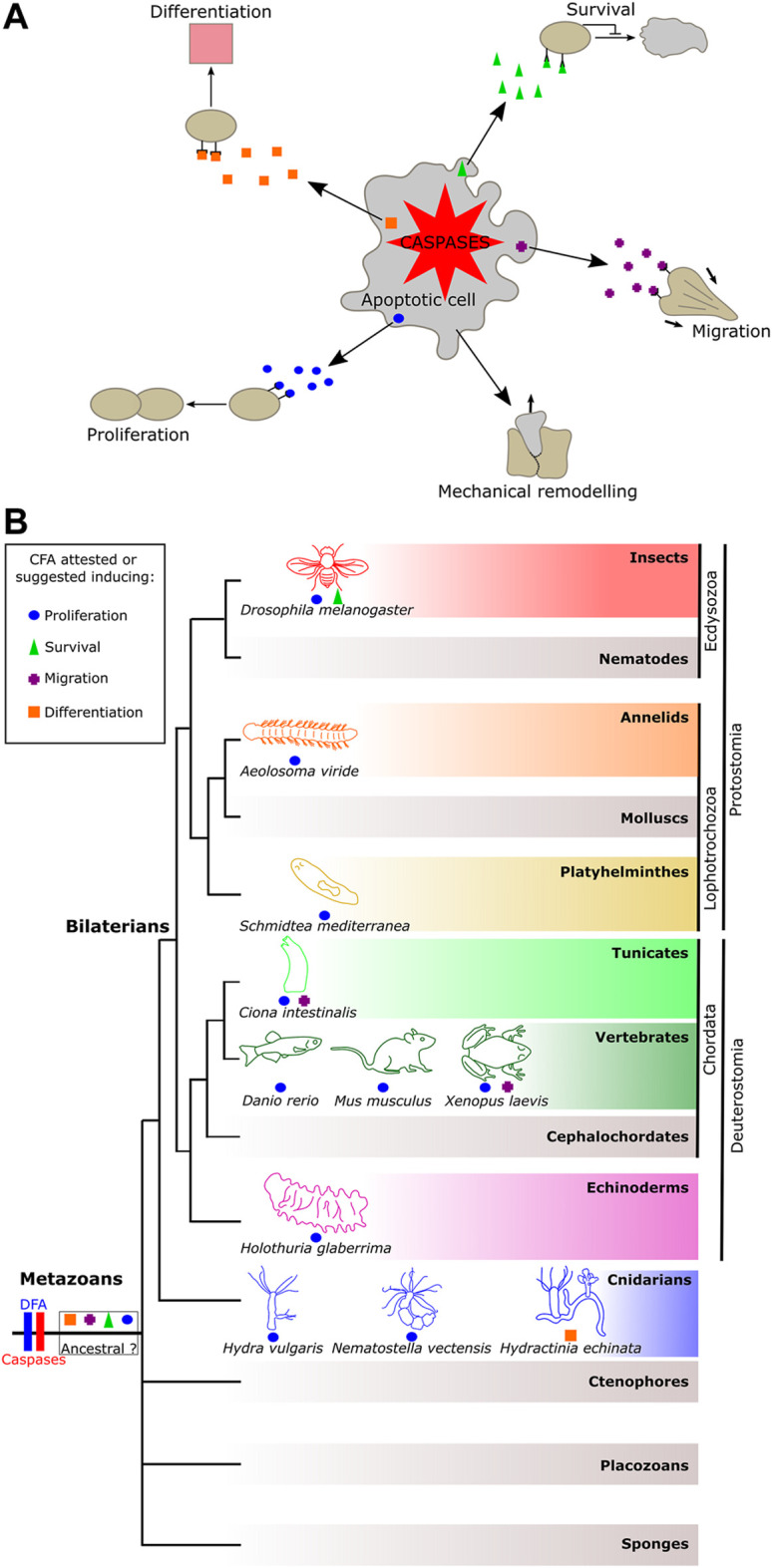
Representation of the constructive function of apoptosis and its occurrence among metazoans. **(A)** Constructive function of apoptosis (CFA) allows apoptotic cells to promote cell remodeling, fates, and behaviors to their neighbor in a caspase-dependent manner. **(B)** Distribution of the CFA among metazoan phyla. The CFA is quite widely distributed, suggesting two possible scenarios: a shared and ancestral origin among animals or conversely several convergent emergences.

However, recent research from different systems has converged into a more complex model of apoptosis during morphogenetic processes with a role beyond the elimination of cells (Fogarty and Bergmann, 2015). Accumulating evidence shows that, while they die, apoptotic cells can trigger tissue remodeling or signal to cells in their vicinity, thereby modulating neighboring cells’ behavior (e.g., migration and proliferation), or fate (e.g., survival and differentiation) in a number of animals (Fogarty and Bergmann, 2015). Hence, in addition to dying, apoptotic cells play non-destructive roles by emitting molecular signals to influence adjacent cells. Until now, varying terminologies have been used in the literature to name specific examples, but no global view exists. We propose to group these phenomena as the “constructive function of apoptosis (CFA),” defined as “any molecular or mechanical cues emitted by an apoptotic cell in a caspase-dependent manner, destined to adjacent cells to induce mechanical remodeling, survival, proliferation, migration, or differentiation” (Figure 1A).

The CFA plays a key function in morphogenetic processes and consequently may be crucial in various human pathologies. The comprehension of CFA is fundamental, considering that apoptosis deregulation can lead to pathologies in humans such as cancer or autoimmune or neurodegenerative diseases.

## The constructive function of apoptosis: State of the art

Apoptosis can change the mechanical properties of a tissue, leading to its remodeling, a phenomenon named “tissue remodeling” or “apoptotic force” (Toyama et al., 2008; Monier et al., 2015). For example, accumulation of the myosin-II filament in an apoptotic cell induces an increase in tissue tension during leg morphogenesis in *Drosophila*, resulting in epithelium folding by pulling live adjacent cells together at the same time as apoptotic cell expulsion (Monier et al., 2015). Similar mechanisms have been observed in the context of dorsal closure during *Drosophila* embryogenesis (Toyama et al., 2008).

Apoptotic cells can induce attraction in a caspase-dependent manner, suggesting an ability to promote migration. This capacity was demonstrated for the first time in nematodes. It acts *via* a phosphatidylserine signal present at the cell surface of apoptotic cells. An apoptotic cell-dependent attractive signal was also demonstrated in mammals (named the “eat-me signal” or “come-and-get-me signal”) (Reddien and Horvitz, 2000; Lauber et al., 2003; Ravichandran, 2003; Ravichandran, 2010). In addition, the migration of primordial germ cells during the metamorphosis of the ascidian *Ciona intestinalis* depends on caspases and is correlated with the speed of apoptotic wave propagation during tail regression, suggesting apoptotic-dependent migration (Krasovec et al., 2019; Krasovec et al., 2021a).

In *Drosophila*, irradiated cells undergoing apoptosis expressed Pvf1, triggering *ban* expression in live adjacent cells *via* activation of their tyrosine–protein kinase receptor Tie-1. Followed by a decrease in caspase expression, this induction confers cell death resistance in case of second irradiation, a mechanism named “the Mahalaki effect” by Bilak et al. (2014).

In addition to survival signal induction, apoptotic cells are able to promote compensatory proliferation (Huh et al., 2004; Wells et al., 2006; Gauron et al., 2013). In *Drosophila*, the caspases Dronc and DrICE coordinate cell death together with cell proliferation during eye and wing development. Thus, an equilibrate balance allows the replacement of dead cells through stimulation of proliferation (Fan and Bergmann, 2008). It appears that this mechanism results from the ability of apoptotic cells to induce an upregulation of mitogen factors such as decapentaplegic and wingless mitogens (Pérez-Garijo et al., 2004; Ryoo et al., 2004). Apoptotic-dependent proliferation has also been reported or suggested in several organisms after injuries (Tseng et al., 2007; Donato-Santana et al., 2008; Mashanov et al., 2010; Pellettieri et al., 2010; Fok et al., 2020). After bisection of the cnidarian *Hydra vulgaris*, the apoptotic cells from the site of injury induce Wnt3 signaling in a caspase-dependent manner, leading to the proliferation of adjacent stem cells, allowing tissue regeneration (Chera et al., 2009). Similar observations were made during the regeneration of the ascidian *C. intestinalis* (Jeffery and Gorički, 2021), and in the context of murine liver regeneration, the authors named this capacity the “phoenix rising” (Li et al., 2010).

Finally, apoptotic cells seem capable of promoting differentiation. During the metamorphosis of the cnidarian *Hydractinia echinata*, the occurrence of caspase-dependent apoptosis in the anterior region of the metamorphosing planula (larval stage) is a prerequisite for the subsequent formation of the stolon region (Wittig et al., 2011).

According to the aforementioned examples and literature, it seems clear that apoptosis can have an instructive function embedded with its classical role of cell elimination. Their regrouping under the CFA gives rise to a unified characterization of these newly reported functions of apoptosis and offers a coherent working framework.

## Discussion

The cleavage capacity of caspases mediates the DFA and is known to be responsible for the morphological aspect of apoptosis. Moreover, according to the aforementioned examples and the literature, caspases could be the key to activating molecular cues, leading to CFA as well. Consequently, we hypothesize that both functions are not exclusive, or rather, they are linked and act together in the global morphogenetic functions of apoptosis.

### Does the constructive function of apoptosis lead to cancer therapy resistance?

During recent decades, an accumulation of studies highlight that apoptosis is able to induce compensatory proliferation after cancer therapy (Wong, 2011; Lagadec et al., 2012; Yang et al., 2014). Tumors, resulting from cancer, are predominantly treated using methods such as irradiation or cytotoxic therapy to kill malignant cells. Unfortunately, these treatments sometimes have poor efficacy, or in the worst case, a counterproductive effect. After treatment, the dying cells (apoptotic cells) can secrete mitogen factors to their neighbors, leading to tumor repopulation (Ng et al., 2013; Morana et al., 2022). For example, caspase-3 and -7 can activate prostaglandin E2 and calcium-insensitive phospholipase A2, respectively, which stimulate cell proliferation. Named “apoptosis-induced proliferation” in the literature (Fogarty and Bergmann, 2017), this undesirable capacity fits into what we defined here as a CFA, especially the proliferation induced by apoptotic cells. Understanding the CFA should be of primary interest in developing curative therapies against cancer that avoid inducing unwanted secondary cell proliferation, which could give rise to more aggressive tumors. Finally, the repopulation that can start within a relatively short time post-therapy could be prevented by a revision of the medical strategy, such as prolonged radiation (Huang et al., 2012).

### The evolution of the morphogenetic function of apoptosis

Although the DFA is documented in almost all metazoan phyla, the CFA has only been described or suggested in a few animals (Figure 1B). These occurrences among metazoans raise the question of whether the sharing of the CFA reflects a common ancestral origin, or, conversely, if it results from several independent evolutionary innovations. According to current knowledge, there is no type of CFA that prevails among metazoans, suggesting several novel acquisitions of the CFA. However, this statement could result from a limited global view due to the imbalance between the high number of animals where the DFA was observed as opposed to the few examples of the CFA reported so far.

Among all types of CFA, apoptosis-induced proliferation is the most documented and was demonstrated in models such as cnidarians, tunicates, and mammals, in addition to being suggested in a few other models, such as planarians and echinoderms (Figure 1B). The over-representation of apoptosis-induced proliferation may result from the fact that mechanisms of regeneration draw much attention in research as their understanding has medical relevance. Also, this role represents a significant innovation by likely improving the chances of survival of an injured animal, certainly leading to its maintenance during evolution due to evolutionary pressure, irrespective of the number of acquisitions of the function.

Understanding the mechanisms of the morphogenetic functions of apoptosis necessitates working on a biological model where apoptosis and other cell fates and behaviors occur simultaneously, such as during metamorphosis or regeneration. In addition, focusing on a biological model with a key phylogenetic position is crucial to explore the evolution of the molecular network underlying both the morphogenetic functions of apoptosis during metazoan diversification.

### Concluding remark

The global comprehension of the morphogenetic function of apoptosis is fundamental because this regulated cell death is ubiquitous in animals. Moreover, apoptosis deregulation can lead to many pathologies in humans, including cancer and autoimmune or neurodegenerative diseases. In light of the CFA, research areas can be investigated to better understand the effect of apoptotic cells on their environment, thus leading to a better understanding of development and disease. Among other examples, the characterization of the CFA could help understand malignant cell survival or the worsening of degenerative diseases.
